# An Unusual Case of Isolated Acute Aphasia in Multiple Sclerosis

**DOI:** 10.7759/cureus.18278

**Published:** 2021-09-25

**Authors:** Hasham Saeed, Qirat Jawed, Muhammad Atif Masood Noori, Syed Hamza Bin Waqar, Aiman Rehan

**Affiliations:** 1 Internal Medicine, Rutgers Health/Trinitas Regional Medical Center, Elizabeth, USA; 2 Internal Medicine, NYC Health + Hospitals/Kings County, New York, USA; 3 Internal Medicine, Veterans Affairs Harbor Health Care, New York, USA; 4 Internal Medicine, State University of New York Downstate Health Sciences Center, New York, USA; 5 Internal Medicine, Dow University of Health Sciences, Karachi, PAK

**Keywords:** ms flare, periventricular plaques, demylinating disorder, transcortical motor aphasia, motor aphasia, multiple sclerosis flare-ups, multiple sclerosis

## Abstract

Acute flare of multiple sclerosis usually presents with sensorimotor deficits in limbs or one side of the face, optic neuritis, internuclear ophthalmoplegia, and/or cerebellar signs and symptoms. Isolated aphasia is observed only in a handful of cases. Herein, we present a case of a patient who presented with isolated transcortical motor aphasia. Initial thought was that the patient was having a cerebrovascular accident as he had a history of uncontrolled hypertension. It was only later found on magnetic resonance imaging (MRI) of the brain that the patient had demyelinating lesions compatible with his new symptoms. He exhibited an excellent response to intravenous methylprednisolone therapy and was discharged with outpatient evaluation for immunotherapy.

## Introduction

Multiple sclerosis (MS) is the most common immune-mediated inflammatory demyelinating disease of the central nervous system. The disease usually manifests itself in the form of acute flares, which can be intermittent, with partial or complete recovery of physical and mental functions (relapsing-remitting), or functions can be affected on a regular basis and may worsen over time (primary progressive). Apart from the classic findings of sensorimotor deficits, optic neuritis, and/or cerebellar findings, neuropsychiatric manifestations in an acute MS flare are usually related to deficits in executive functioning, memory, attention, visuoconstruction, and information processing [1]. Deficits in higher cognitive functions (e.g. language) are rarely observed in patients with MS. Language should be distinguished from speech; the former is the cognitive ability to communicate and manipulate symbols while the latter is a motor ability. Only a handful of cases of language deficits (e.g. aphasia) have been reported to date since 1983 [2]. Here we describe a case of a patient who presented with isolated transcortical motor aphasia with demyelinating lesions compatible with his new symptoms as demonstrated on magnetic resonance imaging (MRI).

## Case presentation

The patient is a 33-year-old African American male with a past medical history of hypertension who was brought to the emergency department for the evaluation of inability to speak. As per family, symptoms started two days before the presentation and progressed over time. He is noncompliant with his blood pressure medications. Blood pressure on arrival was 189/137. Physical examination showed a muscle strength of 5/5 in all four extremities, intact sensations, and normal deep tendon reflexes. There was no appreciable nystagmus. Speech evaluation revealed significant anomia, word-finding difficulty, nonfluency in speech, appropriate comprehension, and slightly reduced attention, requiring occasional repetition of prompts. Obvious limitation of expressive language skills was noted. The patient was only able to reply to questions few hours after presentation with "YES" or "NO." At this point, he denied any recent episodes of headache, nausea, balance problems, falls, or weakness. Initial computed tomography (CT) scan of the head and CT angiography of the head and neck were unremarkable. While waiting for the MRI of the brain, patient was admitted in intensive care unit for neurological examination every hour and management of possible hypertensive emergency/cerebrovascular accident (CVA) for which he was started on nicardipine drip for targeted blood pressure goal, together with aspirin and atorvastatin. He was not a candidate of fibrinolytic therapy (tissue plasminogen activator).

**Figure 1:**
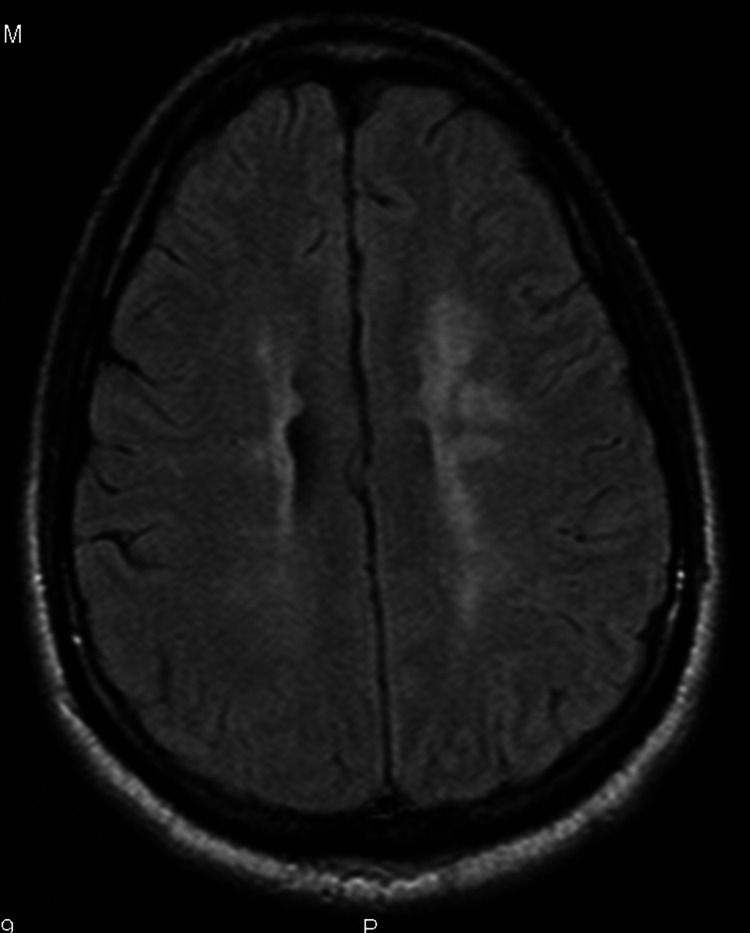
Periventricular hyperintensities more prominent near left frontal region

**Figure 2:**
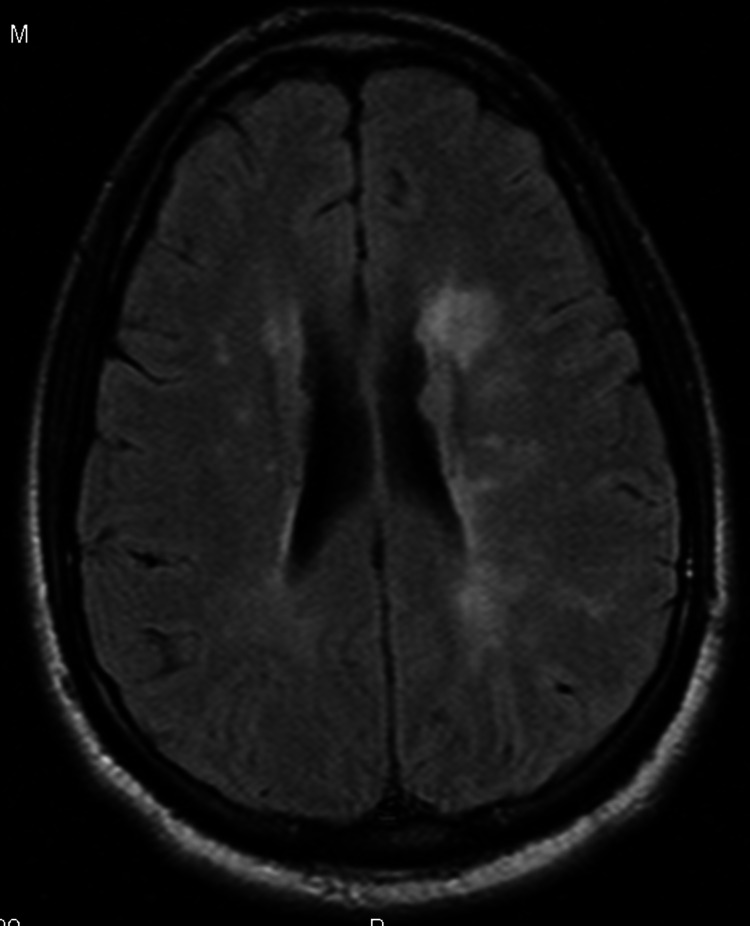
The same periventricular hyperintensities continuing at a lower level

MRI of the brain with contrast showed patchy areas of T2 hyperintense signal within the central aspect of pons and throughout the supratentorial white matter in a periventricular and subcortical distribution (Figures 1, 2). There was also a mild T2 hyperintense signal along the undersurface of the corpus callosum.

MRI spine was negative for any demyelinating plaques. The findings were discussed with the patient and his family and the past history of any neurologic problems was asked. The patient’s mother reported that the patient has been following up with an ophthalmologist for “blurry vision from the left eye” that started in 2019 and has improved significantly since then. He uses contact lenses. A provisional diagnosis of MS was made based on revised McDonald’s criteria (two clinical attacks and clinical evidence of lesions), so we deferred lumbar puncture at this time. The patient was started on methylprednisolone 1 g intravenous infusion daily for five days. Physical, occupational, and speech therapies were initiated. The symptoms improved significantly over the course of four days. He was able to articulate without difficulty and word-finding pauses disappeared over time and he was discharged home on day 5 of admission to the hospital. Follow-up care was established at St. Barnabas Medical Center Immunotherapy Clinic.

## Discussion

Aside from trauma, MS is the most common cause of permanent disability in young adults (such as from spastic paraparesis or cerebellar ataxia) [3]. It is more common in females, with the estimated female-to-male ratio from 1.4:1 to 2.3:1 [4]. The mean age of onset is 18-40 years [5]. The incidence is higher in Europe, northern United States, and southeast Australia [6]. It was thought to be explained in part by ethnicity, with white populations being more susceptible. However, subsequent studies demonstrated an increased incidence in Black adults, suggesting that racial susceptibility may need revision [7]. Our patient is an African-American male and is of the age group in which we typically see the onset of MS.

The classic presentation of MS usually involves sensorimotor deficits in limbs or one side of the face, visual disturbances ranging from optic neuritis and internuclear ophthalmoplegia to visual loss, and/or cerebellar signs and symptoms, i.e., ataxia, nystagmus, and vertigo [8,9]. Cortical syndromes, particularly isolated transcortical aphasia, are exceedingly uncommon [2,10]. Our patient only presented with acute anomic aphasia, nonfluency in speech but appropriate comprehension, and slightly reduced attention span. Repetition was assessed the next day and he could repeat words with some difficulty. He also demonstrated echolalia. These findings, to most extent, are consistent with anterior transcortical motor aphasia. No frank sensorimotor deficits or changes in reflexes were noted.

The course of disease is mostly relapsing-remitting; however, up to 15% of MS cases can have progressive neurologic disability independent of flares, termed as primary progressive [11]. Our patient most likely had relapsing-remitting type, as he mentioned having visual deficits in his left eye in 2019, which could possibly be the first MS flare that was missed. Though long-term follow-up will better be able to classify the course of disease in our patient.

The characteristic neuropathologic lesions of MS, i.e., focal demyelinated plaques with variable degrees of inflammation and gliosis, and partial preservation of axons [12] tend to be located in the optic nerves, spinal cord, brainstem, cerebellum, and the juxtacortical and periventricular white matter [13]. Though uncommon, the lesions can also be found in the corpus callosum [14] and cortical gray matter [15]. Our patient had lesions along the undersurface of the corpus callosum and within the central aspect of pons and throughout the supratentorial white matter in a periventricular and subcortical distribution. The clinical presentation in our patient could be explained to most extent by the lesions in the corpus callosum and fronto-parietal periventricular area. To our knowledge, only few cases of such presentation are reported so far [2,10].

Management of an acute flare of MS revolves around speedy recovery from the attack. The recommendation is the use of high-dose short-term therapy with oral/intravenous glucocorticoids [16]. Our patient had an excellent response to five-day course of 1 g intravenous methylprednisolone daily. He was discharged with the provision of outpatient follow-up for further evaluation and management, as he could be a candidate for disease-modifying therapy. This case, apart from being unique in its presentation, highlights the importance of avoiding anchoring to one diagnosis as this patient with high blood pressure who initially thought to have a CVA was found to have a completely unrelated disorder.

## Conclusions

Isolated abnormalities in higher cognitive functions (e.g. language) without sensorimotor deficits, though uncommon, can be a presenting feature of MS. Detailed history should be obtained from the patient or family for any neurologic symptoms in the past. Contrast-enhanced MRI is the key to diagnosis by visualizing demyelinating plaques. Language impairments can have a major impact on quality of life as well as on the efficiency of rehabilitation. Moreover, MS should always be kept in the differentials for CVA to avoid delay in diagnosis and management and potentially avoid unnecessary harms from fibrinolytic therapy for ischemic CVA. Further studies are required to group together various forms of language impairments (e.g., expressive, sensory, transcortical motor, mixed aphasia) to better be able to categorize language manifestations in different types of MS.
